# Detection of cannabinoid receptors CB1 and CB2 within basal ganglia output neurons in macaques: changes following experimental parkinsonism

**DOI:** 10.1007/s00429-014-0823-8

**Published:** 2014-06-28

**Authors:** Salvador Sierra, Natasha Luquin, Alberto J. Rico, Virginia Gómez-Bautista, Elvira Roda, Iria G. Dopeso-Reyes, Alfonso Vázquez, Eva Martínez-Pinilla, José L. Labandeira-García, Rafael Franco, José L. Lanciego

**Affiliations:** 1 Neurosciences Division, Center for Applied Medical Research (CIMA), University of Navarra, Pio XII Avenue 55, 31008 Pamplona, Spain; 2 Network Center for Biomedical Research in Neurodegenerative Diseases (CIBERNED), Madrid, Spain; 3 Department of Neurosurgery, Complejo Hospitalario de Navarra, Pamplona, Spain; 4 Department of Morphological Sciences, University of Santiago de Compostela, Santiago de Compostela, Spain; 5 Department of Biochemistry and Molecular Biology, University of Barcelona, Barcelona, Spain

**Keywords:** GPCRs, Cannabis, Cannabinoid receptor heteromer, Dyskinesia, Globus pallidus, MPTP

## Abstract

Although type 1 cannabinoid receptors (CB_1_Rs) are expressed abundantly throughout the brain, the presence of type 2 cannabinoid receptors (CB_2_Rs) in neurons is still somewhat controversial. Taking advantage of newly designed CB_1_R and CB_2_R mRNA riboprobes, we demonstrate by PCR and in situ hybridization that transcripts for both cannabinoid receptors are present within labeled pallidothalamic-projecting neurons of control and MPTP-treated macaques, whereas the expression is markedly reduced in dyskinetic animals. Moreover, an in situ proximity ligation assay was used to qualitatively assess the presence of CB_1_Rs and CB_2_Rs, as well as CB_1_R–CB_2_R heteromers within basal ganglia output neurons in all animal groups (control, parkinsonian and dyskinetic macaques). A marked reduction in the number of CB_1_Rs, CB_2_Rs and CB_1_R–CB_2_R heteromers was found in dyskinetic animals, mimicking the observed reduction in CB_1_R and CB_2_R mRNA expression levels. The fact that chronic levodopa treatment disrupted CB_1_R–CB_2_R heteromeric complexes should be taken into consideration when designing new drugs acting on cannabinoid receptor heteromers.

## Introduction

The presence of type 1 (CB_1_Rs) and type 2 (CB_2_Rs) cannabinoid receptors in the CNS, particularly the basal ganglia, has fuelled research into their role in motor function and dysfunction. The ubiquitously expressed CB_1_Rs are found predominantly in neurons of the central and peripheral nervous system (Freund et al. 2003). In the rat, CB_1_Rs are found in the striatum, both in GABAergic projection neurons and interneurons (Hohmann and Herkenham 2000; Moldrich and Wenger 2000). Initially detected only in peripheral tissue (Munro et al. 1993; Galiegue et al. 1995; Klein et al. 2003), the presence of CB_2_Rs in the CNS has been somewhat controversial. Present at lower expression levels than CB_1_Rs, differences arising from different staining protocols and complications with negative controls, such as CB_2_R-KO mice have delayed confirmation of the presence of these receptors in the CNS (Munro et al. 1993; Galiegue et al. 1995; Griffin et al. 1999). Nowadays, CB_2_Rs have been reported in microglia (Kearn and Hilliard 1997; Golech et al. 2004; Nuñez et al. 2004; Stella 2004; Maresz et al. 2005; Ashton et al. 2006) and neurons (Skaper et al. 1996; Stander et al. 2005; Van Sickle et al. 2005; Wotherspoon et al. 2005; Beltramo et al. 2006; Onaivi et al. 2006; Brusco et al. 2008a, b; den Boon et al. 2012). Within the basal ganglia, CB_2_Rs are expressed in neurons from both segments of the globus pallidus (GPe and GPi) of *Macaca fascicularis* (Lanciego et al. 2011) and in the substantia nigra *pars reticulata* (SNr) of the rat (Gong et al. 2006).

The expression of cannabinoid receptors in the basal ganglia has important implications for motor dysfunction, such as Parkinson’s disease (PD). Data suggest that CB_1_R expression levels in the striatum are upregulated in rodent and primate models of PD (Mailleux and Vanderhaeghen 1993; Romero et al. 2000; Lastres-Becker et al. 2001) and in PD patients (Lastres-Becker et al. 2001). Activation of CB_1_Rs inhibits neurotransmitter release through endocannabinoid retrograde signaling, capable of reducing neuronal signaling (Shen et al. 1996). In basal ganglia output nuclei (GPi and SNr), CB_1_R activation reduces both GABA and glutamate release from striatal and subthalamic inputs, respectively (Sañudo-Peña et al. 1999). Intracellular CB_2_Rs have also been suggested to reduce neuronal firing rate (den Boon et al. 2012). Despite the interest related to endocannabinoid-based neuroregulation in the basal ganglia, neuronal CB_1_R and CB_2_R expression has not been properly characterized within GPi and SNr.

The present study was conducted to establish whether CB_1_R and CB_2_R transcripts are present in pallidothalamic projection neurons. Given that mRNA for the two receptors was present in the same neurons, and an in situ proximity ligation assay (PLA) detected the localization of CB_1_Rs, CB_2_Rs and CB_1_R–CB_2_R heteromers in the cell somata of projection neurons. Experiments performed in control, parkinsonian and dyskinetic macaques demonstrated that CB_1_Rs, CB_2_Rs and CB_1_R–CB_2_R heteromer levels were similar in naïve and parkinsonian animals, and markedly reduced in dyskinetic macaques.

## Materials and methods

A total of eight naïve adult male *Macaca fascicularis* primates (body weight 3.8–4.5 kg) were used in this study. Animal handling was conducted in accordance with the European Council Directive 86/609/EEC, as well as in agreement with the Society for Neuroscience Policy on the Use of Animals in Neuroscience Research. The experimental design was approved by the Ethical Committee for Animal Testing of the University of Navarra (ref: 009-12). All animals were captive-bred and supplied by Harlan Laboratories.

### MPTP treatment and levodopa

The dopaminergic neurotoxin 1-methyl-4-phenyl-1,2,3,6-tetrahydropyridine (MPTP; Sigma) was administered intravenously to four macaques at a concentration of 0.2 mg/kg (injected once weekly) until animals reached a stable parkinsonian syndrome. The severity of MPTP-induced parkinsonism was evaluated by two independent blind observers using clinical rating scales (Kurlan et al. 1991) where the highest score was 29. All MPTP-treated macaques reached a stable score between 21 and 25 points that was maintained over a period of 2 months of MPTP washout. Two monkeys were selected to receive daily oral treatment with levodopa and benserazide (25 mg/kg of Madopar, Roche, France). These monkeys developed a mild dyskinetic syndrome by the end of the first month of treatment, then displaying overt dyskinetic symptoms 1 month later and remained stable until the CTB retrograde tracer injection. The extent of the MPTP-induced dopaminergic depletion was confirmed by immunohistochemical detection of tyrosine hydroxylase, as shown in Rico et al. (2010) and Conte-Perales et al. (2011).

### Stereotaxic surgery, perfusion and tissue processing

Surgical anesthesia was induced by intramuscular injection of ketamine (5 mg/kg) and midazolam (5 mg/kg), resulting in deep anesthesia over a period of 2–3 h. Local anesthesia was implemented just before surgery by means of a 10 % solution of lidocaine. Analgesia was achieved with a single intramuscular injection of flunixin meglumine (Finadyne, 5 mg/kg) delivered at the end of the surgical procedure and repeated 24 and 48 h post surgery. A similar schedule was followed for antibiotic delivery of ampicillin (0.5 ml/day). After surgery, animals were kept under constant monitoring in single cages with ad libitum access to food and water.

Stereotaxic coordinates for ventral anterior and ventral lateral thalamic nuclei (VA/VL) were taken from the atlas by Lanciego and Vázquez (2012). During surgery, target selection was assisted by ventriculography. Selected coordinates for targeting VA/VL with cholera toxin B subunit (CTB) were 4.5 mm caudal to the anterior commissure (ac), 4 mm lateral to the midline and 2 mm dorsal to the intercommissural plane (ac–pc line).

Six monkeys (2 control, 2 parkinsonian and 2 dyskinetic monkeys) received a single pressure injection of 5 µl of unconjugated cholera toxin subunit B (CTB, List Biological Laboratories, Campbell, CA) through a Hamilton syringe (5 mg/ml in 0.01 M phosphate buffer, pH 7.5) in the VA/VL nuclei. Tracer delivery was accomplished in pulses of 1 µl every 2 min and, once completed, the microsyringe was left in place for 15 min before withdrawal to minimize tracer uptake through the injection tract.

After 2 weeks of postsurgery, animals were anesthetized with an overdose of 10 % chloral hydrate and perfused transcardially (for dyskinetic monkeys, terminal anesthesia was administered at the time point at which they showed overt, peak-of-dose dyskinesias). The perfusates consisted of a saline Ringer solution followed by 3,000 ml of a fixative solution containing 4 % paraformaldehyde and 0.1 % glutaraldehyde in 0.125 M phosphate buffer (PB), pH 7.4. Perfusion was continued with 1,000 ml of a cryoprotectant solution containing 10 % glycerin and 1 % dimethylsulphoxide (DMSO) in 0.125 M PB, pH 7.4. Once perfusion was completed, the skull was opened, the brain removed, and stored for 48 h in a cryoprotectant solution containing 20 % of glycerin and 2 % DMSO in 0.125 M PB, pH 7.4. All solutions used for fixation and cryoprotection were treated with 0.1 % diethylpyrocarbonate (DEPC) and autoclaved prior to their use. Finally, frozen serial sagittal sections (40 µm-thick) were obtained on a sliding microtome and collected in 0.125 M PB, pH 7.4, as 15 series of adjacent sections. The series were used for: (1) immunohistochemical detection of tyrosine hydroxylase, (2) immunohistochemical detection of transported CTB, later counterstained with Nissl stain, (3) single colorimetric in situ hybridization to detect CB_1_R mRNA, (4) single colorimetric in situ hybridization to detect CB_2_R mRNA, (5) immunofluorescent detection of transported CTB combined with dual fluorescent in situ hybridization using antisense riboprobes for CB_1_R and CB_2_R, (6) immunofluorescent detection of CTB combined with in situ proximity ligation assay (PLA) to detect CB_1_Rs, (7) immunofluorescent detection of CTB combined with PLA to detect CB_2_Rs and (8) immunofluorescent detection of transported CTB combined with PLA to detect CB_1_R–CB_2_R heteromers. Some additional sections were used for the ultrastructural detection of CB_1_R–CB_2_R heteromers, as indicated below. The remaining series of sections were stored at −80 °C for further histological processing, if needed.

### Detection of transported CTB

Immunohistochemical detection of transported CTB was carried out on sagittal sections throughout the entire mediolateral extent of the left brain hemisphere. Sections were incubated with a primary antibody against CTB raised in rabbit (1:2000; overnight at 4 °C; GenWay, San Diego, CA, USA) followed by a biotinylated donkey antirabbit IgG (1:200; 2 h at room temperature—RT; Jackson Immunoresearch). Sections were incubated in HRP-conjugated streptavidin (1:5000; 90 min at RT: Sigma) and finally visualized in brown with DAB (Sigma). Sections were mounted on gelatin-coated glass slides, dried at RT and subsequently counterstained with thionin to accurately delineate the boundaries of the brain structures showing CTB labeling. Once the Nissl stain was completed, sections were coverslipped with Entellan (Merck).

### Polymerase chain reaction

For PCR amplification, fresh tissue samples (unfixed) from two control naïve primates available in our monkey brain bank were used. Briefly, a brain block containing the striatum, GPe and GPi was frozen rapidly in isopentane, cooled with liquid nitrogen and coronal sections (20 μm thick) were obtained using a cryostat. The sections were mounted on dedicated plastic-coated slides (Leica Microsystems) for laser-guided capture microdissection (LCM). Under the LCM microscope (Leica), the boundaries of the striatum, GPe and GPi were delineated and dissected separately from the tissue using the laser beam. The tissue samples obtained from these regions were collected in separate 0.5 ml Eppendorf vials containing lysis buffer for RNA extraction. Total RNA was extracted using the Absolutely RNA Nanoprep kit (Stratagene, La Jolla, CA, USA) according to the manufacturer’s instructions and including the optional DNase I digestion step. The RNA, eluted in a final volume of 10 μl, was used entirely for reverse transcription. The cDNA template was obtained by adding 1 μl 10 mM dNTP mix, 1 μl 0.1 M DTT, 50 ng hexamers, 1 μl RNase inhibitor (40 U/μl; Promega, Madison, WI, USA), 4 μl 5× first-stand buffer, 2 μl sterile water and 1 μl SuperScript III reverse transcriptase (200 U/μl; Invitrogen) in a final volume of 20 μl and incubated at 50 °C for 60 min. Subsequently, the reaction was inactivated by heating at 70 °C for 15 min.

PCRs were carried out in a final volume of 50 μl containing 25 mM of each primer, 0.5 μl of *Taq* DNA polymerase (Bioline), 5 μl 10× *Taq* DNA polymerase PCR buffer, 1.5 μl MgCl_2_, 2 μl dNTP and 8 μl per reaction of pure cDNA for amplification in the case of CB_1_R and CB_2_R and 2 μl of cDNA in the case of the control gene GAPDH. After 94 °C for 5 min, the thermocycling parameters were as follows: 35 cycles of 94 °C for 30 s, 58 °C for 30 s and 72 °C for 1 min. The extension reaction was carried out for 10 min at 72 °C, and reaction products were stored at 4 °C. The primers used in PCR were: forward CATCCAGTGTGGGGAGAACT and reverse TATGGTCCACATCAGGCAAA for CB_1_R (product size 445 bp), forward CATCACTGCCTGGCTCACT and reverse AGCATAGTCCTCGGTCCTCA for CB_2_R (product size 662 bp) and forward CATCCTGCACCACCAACTGCTTAG and reverse GCCTGCTTCACCACCTTCTTGATG for GAPDH (product size 343 bp). The PCR products were analyzed by electrophoresis on a 1 % agarose gel containing SYBR Safe DNA gel stain (Invitrogen) under ultraviolet light.

### Synthesis of sense and antisense riboprobes for CB_1_R and CB_2_R mRNA

Total RNA was isolated from a *Macaca fascicularis* using the Trizol reagent (Invitrogen Life Technologies, Carlsbad, CA, USA). Spleen tissue samples were disrupted in 1 ml Trizol reagent using a homogenizer. After 5 min incubation at RT, 0.2 ml of chloroform was added and mixed vigorously; the sample was then centrifuged at 12,000*g* for 15 min at 4 °C. Following centrifugation, the supernatant was placed in a new tube, and 0.5 ml isopropanol was added followed by incubation for 10 min at RT. The RNA pellet was obtained by centrifugation at 12,000*g* for 10 min at 4 °C. The pellet was washed in 1 ml 75 % ethanol and, after vaporization of ethanol, dissolved in 30 ml DEPC-treated water. Absorbance at 260 nm was determined to quantify the amount of total RNA, which was stored at −80 °C.

First-strand cDNA was synthesized from the total RNA extracted and 0.5 mg of total RNA was subjected to PCR by adding Superscript III reverse transcriptase (Invitrogen) (1 µl, 200 U/µl), oligo-(dT) (1 ml, 50 mM), buffer (4 µl, 5× First-Strand Buffer: 200 mM Tris–HCl, 500 mM KCl, 50 mM MgCl_2_), dithiothreitol (1 µl, 0.1 M) and mixed dNTPs (1 µl, 10 mM; Invitrogen) adding DEPC-treated water to make up a final volume of 20 µl.

Template cDNA sequences were obtained from GenBank (http://www.ncbi.nlm.nih.gov/). Oligonucleotide primers were designed using Primer3Input v.0.4.0 software (http://www.frodo.wi.mit.edu/cgi-bin/primer3/primer3_www.cgi). Primers designed for CB_1_R and CB_2_R were the abovementioned primers. PCR was performed with Pfx polymerase (Invitrogen) and 35 cycles of amplification (denaturation at 95 °C for 1 min, annealing at 58 °C for 30 s, extension at 68 °C for 1 min) and a final extension at 68 °C for 10 min. The PCR products were analyzed by electrophoresis on a 0.8 % agarose gel containing SYBR Safe DNA gel stain (Invitrogen) under ultraviolet light and purified using a QIAquick Gel Extraction kit (QIAGEN GmbH).

The PCR product was later inserted into the plasmid vector (pCR-Blunt II-TOPO; Invitrogen) and used to transform competent *E. coli* cells (Invitrogen). The product extracted using the Miniprep kit (Qiagen) was then sequenced (3130XL Genetic Analyzer, Applied Biosystems). The computer-assisted homology searches (see http://www.blast.ncbi.nlm.nih.gov/Blast.cgi) conducted showed that the CB_1_R cDNA sequence had 100 % homology with human CB_1_R transcript variant 1 (accession number NM_016083) and variant 2 (accession number NM_033181), and 99 % homology with *Macaca mulatta* CB_1_R (accession number NM_001032825). CB_2_R cDNA sequence had 94 % homology with human CB_2_R (NM_001841) and 99 % homology with *Macaca mulatta* CB_2_R (accession number XM_001105018) sequences, without any significant homology with CB_1_R for the different species, and the same holds true when comparing the homologies of CB_1_R cDNA sequence with CB_2_R. Furthermore, the designed probe recognizes both CB_2A_R and CB_2B_R isoforms which have been recently reported (Liu et al. 2009).

Sense and antisense riboprobes for *Macaca fascicularis* CB_1_Rs or CB_2_Rs were transcribed from the Zero Blunt TOPO PCR cloning kit plasmid. The plasmid was linearized and the sense or antisense probes were transcribed with the appropriate RNA polymerases (Boehringer Mannheim, Germany). The transcription mixture included 1 µg template plasmid, 1 mM each of ATP, CTP and GTP, 0.7 mM UTP and 0.3 mM digoxigenin-UTP, 10 mM DTT, 50 U RNase inhibitor and 1 U of either T7 or SP6 RNA polymerase in a volume of 50 µl. After 2 h at 37 °C, the template plasmid was digested with 2 U RNase-free DNAse for 30 min at 37 °C. The sense and antisense riboprobes were then precipitated by the addition of 100 µl of 4 M ammonium acetate and 500 µl of ethanol and finally recovered by centrifugation at 4 °C for 30 min. The quality of the synthesis was monitored by dot blot.

### Dual fluorescent in situ hybridization combined with immunofluorescent detection of transported CTB

Dual fluorescent in situ hybridization procedures were carried out on free-floating sections that were incubated twice in 0.1 % DEPC in PB for 15 min and pre-equilibrated for 10 min in 5× SSC (0.75 M NaCl, 0.0075 M Na–citrate). Sections were then incubated at 58 °C for 2 h in a hybridization solution containing 50 % deionized formamide, 5× SSC and 40 µg/µl of denatured salmon DNA in H_2_O-DEPC. A mixture of the biotin-labeled CB_2_R riboprobe and digoxigenin-labeled CB_1_R riboprobe were used, denatured for 5 min at 77 °C and then added to the hybridization mix at 400 ng/ml. Sections were hybridized in this solution overnight at 58 °C. Posthybridization washes were carried out in 2× SSC at RT for 15 min, 2× SSC for 30 min at 65 °C and then in 0.1× SSC for 30 min at 65 °C.

The biotin-labeled probe was the first to be visualized after immersing the sections for 15 min in 3 % H_2_O_2_ to inactivate the endogenous peroxidase activity. After several rinses in TNT buffer (0.1 M Tris–HCl, pH 7.5, 0.15 M NaCl, 0.05 % Tween 20) the sections were equilibrated for 30 min in TNB (0.1 M Tris–HCl, pH 7.5, 0.15 M NaCl, 0.5 % blocking reagent, Perkin Elmer), then incubated with streptavidin-conjugated horseradish peroxidase (1:50, Perkin Elmer) in TNB buffer for 30 min at RT. After several washes with TNT buffer, the sections were incubated for 10 min in biotinyl tyramide (1:50 in amplification diluent; Perkin Elmer). The fluorescent labeling was then visualized using Alexa-633 conjugated streptavidin (1:100; Molecular Probes).

The CB_1_R mRNA transcript, detected with a digoxigenin-labeled riboprobe, was visualized immediately following the biotin-labeled probe. Sections were briefly rinsed with TN buffer (0.1 M Tris–HCl, pH 7.5, 0.15 M NaCl) and incubated for 90 min at RT with an anti-digoxigenin antibody raised in sheep (1:1200; Roche Diagnostics). After several rinses in TNT buffer, sections were washed three times for 5 min with TNM buffer (0.1 M Tris–HCl, pH 8, 1 M NaCl, 100 mM MgCl_2_) at RT and transcripts were finally visualized using the HNPP fluorescence detection kit (Roche Diagnostics), to be viewed with the red channel.

Immediately following the double fluorescent in situ hybridization assay, fluorescent immunodetection of transported CTB was carried out. As outlined above, a rabbit anti-CTB primary antibody was used, followed by a secondary donkey antirabbit Alexa^®^488-conjugated antibody (1:200, 2 h; Molecular Probes). Sections were then mounted on gelatin-coated glass slides, dried in the dark, dehydrated rapidly in toluene and coverslipped with DPX (VWR International).

### Fusion proteins and expression vectors

Human cDNA for CB_1_, CB_2_ and dopamine D_4.2_ receptors cloned in pcDNA3.1 were amplified without their stop codons using sense and antisense primers harboring either unique *Eco*RI and *Bam*H1 sites (CB_1_R, CB_2_R) or* Xho*1 and *Eco*R1 (D_4.2_R). The fragments were then subcloned to be in-frame with Rluc into the *Eco*RI and *Bam*H1 (CB_1_R) restriction site of an Rluc-expressing vector (pRluc-N1, PerkinElmer, Wellesley, MA), or into the *Bam*H1 and *Eco*RI (CB_2_R) or *Xh*o1 and *Eco*R1 (D_4.2_R) restriction site of an EYFP expressing vector (EYFP-N1; enhanced yellow variant of GFP; Clontech, Heidelberg, Germany), to create plasmids that express CB_1_R, CB_2_R or D_4.2_R fused to Rluc or YFP on the C-terminal end of the receptor (CB_1_R–Rluc, CB_2_R-YFP or D_4.2_R-YFP). The expression of constructs was tested using confocal microscopy and receptor functionality using the ERK1/2 activation pathway.

### Cell line cultures and transfection

Human embryonic kidney 293T (HEK-293T) cells were grown in DMEM supplemented with 2 mM l-glutamine, 1 mM sodium pyruvate, 100 units/ml penicillin/streptomycin and 5 % (v/v) heat-inactivated fetal bovine serum (FBS) (all supplements were from Invitrogen, Paisley, Scotland, UK). Cells were maintained at 37 °C in a humidified atmosphere of 5 % CO_2_, and were passaged when they were 80–90 % confluent, i.e. approximately twice a week.

HEK-293T cells were transiently transfected with the corresponding fusion protein cDNA by the ramified PEI (PolyEthylenImine, Sigma, St. Louis, MO, USA) method. Cells were incubated (4 h) with the corresponding cDNA together with ramified PEI (5 ml of 10 mM PEI for each mg cDNA) and 150 mM NaCl in a serum-starved medium. After 4 h, the medium was changed to a fresh complete culture medium. After 48 h of transfection, cells were washed twice in quick succession in Hanks’ balanced salt solution HBSS (137 mMNaCl, 5 mMKCl, 0.34 mM Na_2_HPO_4_ × 12H_2_O, 0.44 mM KH_2_PO_4_, 1.26 mM CaCl_2_ × 2H_2_O, 0.4 mM MgSO_4_ × 7H_2_O, 0.5 mM MgCl_2_, 10 mM HEPES, pH 7.4) supplemented with 0.1 % glucose (w/v), detached by gently pipetting and resuspended in the same buffer. To control the cell number, sample protein concentration was determined using a Bradford assay kit (Bio-Rad, Munich, Germany) using bovine serum albumin dilutions as standards. HEK-293T cell suspension (20 µg of protein) was distributed into 96-well microplates; black plates with a transparent bottom (Porvair, Leatherhead, UK) were used for fluorescence determinations, whereas white opaque plates (Porvair, Leatherhead, UK) were used for bioluminescence resonance energy transfer (BRET) experiments.

### BRET assays

HEK-293T cells were transiently co-transfected with the indicated amounts of plasmid cDNAs corresponding to the indicated fusion proteins (see Fig. 6). To quantify receptor-fluorescence expression, cells (20 µg protein) were distributed in 96-well microplates (black plates with a transparent bottom; Porvair, Leatherhead, UK) and fluorescence was read using a Mithras LB 940 (Berthold, Bad Wildbad, Germany) equipped with a high-energy xenon flash lamp, using an excitation filter of 485 nm. Receptor-fluorescence expression was determined as fluorescence of the sample minus the fluorescence of cells expressing protein-Rluc alone. For BRET measurements, the equivalent of 20 µg of cell suspension were distributed in 96-well microplates (white plates; Porvair, Leatherhead, UK) and 5 µM coelenterazine H (PJK GMBH, Germany) was added. After 1 min of adding coelenterazine H, readings were collected using a Mithras LB 940 (Berthold, Bad Wildbad, Germany) that allows the integration of the signals detected in the short wavelength filter at 485 nm (440–500 nm) and the long wavelength filter at 530 nm (510–590 nm). To quantify the receptor-Rluc expression luminescence readings were performed after 10 min of adding 5 µM coelenterazine H. Cells expressing BRET donors alone were used to determine background. The net BRET is defined as [(long-wavelength emission)/(short-wavelength emission)]−C_f_ where C_f_ corresponds to [(long-wavelength emission)/(short-wavelength emission)] for the Rluc construct expressed alone in the same experiment. BRET curves were fitted using a nonlinear regression equation, assuming a single phase with GraphPad Prism software (San Diego, CA, USA). BRET is expressed as mili BRET units (mBU: 1,000 × net BRET).

### In situ proximity ligation assay (PLA)

The PLA technique was carried out both on cell cultures as well as on histological sections. Briefly, 3 different HEK-293T cell lines transiently expressing CB_1_R, CB_2_R or both receptors were fixed in 4 % paraformaldehyde for 15 min and washed with PBS containing 20 mM glycine to quench the aldehyde groups. The presence/absence of receptor–receptor molecular interaction in these samples was detected using the Duolink II in situ PLA detection kit (Olink Bioscience, Uppsala, Sweden). To detect CB_1_R–CB_2_R heteromers, the rabbit anti-CB_1_R antibody (Thermo Scientific, Rockford, USA) was linked to a plus PLA probe and the rabbit anti-CB_2_R antibody (Cayman Chemical, Ann Arbor, USA) was linked to a minus PLA probe following the manufacturer’s instructions. After incubation for 1 h at 37 °C with the blocking solution in a preheated humidity chamber, cell cultures were incubated overnight with these PLA probe-linked antibodies (final concentration of 65 μg/ml) at 4 °C. Next, samples were immersed for 1 h in a 1:400 solution of TOPRO-3 (Molecular Probes-Invitrogen) for nuclear staining. After washing with buffer A at RT, the cells were incubated with the ligation solution for 1 h at 37 °C in a humidity chamber. Following washes with buffer A, samples were incubated with the amplification solution for 100 min at 37 °C in humidity chamber and then washed with buffer B, followed by another wash with buffer B × 0.01. Samples were mounted using an aqueous mounting medium. Cell lines transfected only with either CB_1_R or CB_2_R were used as appropriate negative control assays for the PLA technique to ensure that there was a lack of nonspecific labeling.

Tissue sections containing the GPi were used for the immunofluorescent visualization of transported CTB followed by a PLA protocol to detect CB_1_Rs, CB_2_Rs, and CB_1_R–CB_2_R heteromers. The PLA technique has been successfully employed to detect G-protein-coupled receptor heteromers in the striatum (Trifilieff et al. 2011) as well as in the globus pallidus (Callen et al. 2012). The method is based on the use of two primary antibodies (against each target receptor) covalently coupled to a pair of affinity oligonucleotide probes (a plus and minus probe). Only when the target proteins are in close proximity (<17 nm) do the probes ligate (Callen et al. 2012) and form templates for rolling circle amplification (amplifying the DNA molecule 1,000-fold) (Söderberg et al. 2008; Trifilieff et al. 2011). Hybridization of complementary fluorescently labeled oligonucleotides with the amplified DNA is then seen as a red dot with fluorescent microscopy, representing a single protein–protein interaction.

The receptor–receptor molecular interaction in these samples was detected using the Duolink II in situ PLA detection kit (Olink Bioscience). To detect CB_1_R–CB_2_R heteromers in tissue sections, the rabbit anti-CB_1_R antibody (Thermo Scientific) was linked to a plus PLA probe and the rabbit anti-CB_2_R antibody (Cayman Chemical) was linked to a minus PLA probe following the manufacturer’s instructions. After incubation for 1 h at 37 °C with the blocking solution in a preheated humidity chamber, tissue sections were incubated overnight with these PLA probe-linked antibodies (final concentration of 65 μg/ml) at 4 °C. After washing with buffer A at RT, sections were incubated with the ligation solution for 1 h at 37 °C in a humidity chamber. Following washes with buffer A, sections were incubated with the amplification solution for 100 min at 37 °C in a humidity chamber. Sections were then washed with buffer B, followed by a wash with buffer B× 0.01. Samples were mounted using an aqueous mounting medium. Appropriate negative control assays were carried out to ensure that there was a lack of nonspecific labeling and amplification. In addition to using the PLA technique for detecting CB_1_R–CB_2_R heteromers, we have modified the original protocol according to the suggestions issued by the supplier in order to further use this technique to detect single cannabinoid receptors. Accordingly, we have used either a rabbit anti-CB_1_R (Thermo Scientific) or a rabbit anti-CB_2_R (Cayman Chemical) followed by two secondary donkey-antirabbit antibodies, one linked to a plus PLA probe, the other linked to a minus PLA probe.

### Confocal visualization settings and densitometries

Stained samples (in situ hybridization and PLA) were inspected under a Zeiss 510 Meta confocal laser-scanning microscope (CLSM). To ensure appropriate visualization of the labeled elements and to avoid false positive results, the emission from the argon laser at 488 nm was filtered through a band pass filter of 505–530 nm and color-coded in green. The emission following excitation with the helium laser at 543 nm was filtered through a band-pass filter of 560–615 nm and color coded in light blue. Finally, a long-pass filter of 650 nm was used to visualize the emission from the helium laser at 633 nm and color coded in red. A similar band-pass filter setup was used for the visualization of either CTB-labeled neuronal structures showing PLA labeling. Since in these cases, there was no need to use the infrared laser, the observed emission from the helium laser at 543 nm was color-coded in red.

### Electron microscopy

Ultrastructural detection of CB_1_R–CB_2_R heteromers was carried out using the PLA technique followed by immunogold labeling and silver enhancement. Proximity probes consisted of affinity-purified antibodies modified by covalent attachment of 5′ end of various oligonucleotides to each primary antibody. To create our PLA probes we conjugate a rabbit anti-CB_1_R with a PLUS oligonucleotide (Sigma, Duolink^®^ In Situ Probemaker PLUS catalogue number DUO92009) and a rabbit anti-CB_2_R with a MINUS oligonucleotide (Sigma, Duolink^®^ In Situ Probemaker MINUS catalogue number DUO92010) following the manufacturer’s instructions.

Free-floating sections were incubated 15 min in a 0.1 % sodium borohydride solution, after rinsing in PB and buffer A (Wash buffer A catalogue number DUO82047, Sigma) were incubated for 1 h at 37 °C with the blocking solution (Sigma, Duolink^®^ In Situ Probemaker PLUS catalogue number DUO92009), followed by overnight incubation with the PLA probe-linked antibodies described above (final concentration of 60 µg/ml) at 4 °C. The presence/absence of receptor–receptor molecular interaction in these samples was detected using the Duolink II in situ PLA detection kit (Sigma, Duolink^®^ In Situ Detection Reagents Brightfield, catalogue number DUO92012). Following the detection protocol described by the manufacturer, sections were washed with buffer A at room temperature and incubated with the ligation solution for 1 h at 37 °C. Following washes with buffer A, samples were incubated with the amplification solution for 100 min at 37 °C. Afterwards sections were rinsed in buffer A and incubated the detection solution, consisting of horseradish peroxidase (HRP) labeled oligonucleotides for 1 h at room temperature. After rinsing in buffer A, free-floating sections were incubated in blocking solution containing 3 % NGS, 0.005 % triton X-100, 1 % BSA, 0.05 M glycine and 1 % w/v nonfat dry milk in PBS for 1 h. Afterwards sections were incubated overnight at 4 °C with goat antihorseradish peroxidase 4 nm colloidal gold (Jackson Immunoresearch, catalogue number 123-185-021) 1:100 diluted in a solution of 3 % NGS, 0.005 % triton X-100, 1 % BSA and 1 % w/v nonfat dry milk in PBS.

Sections were washed with PB 0.1 M and postfixed in a 2.5 % glutaraldehyde solution for 2 h. Washes with PB 0.1 M were followed by washes with distilled water and finally sections were incubate in a silver enhancement solution (Aurion R-Gent SE-EM Silver Enhancement Reagents, catalogue number 500.044) for 90 min at room temperature. After rinsing with distilled water sections were postfixed in 1 % Osmium solution in distilled water for 20 min. Rinse in 0.1 M PB and dehydrate 2 × 10 min in 50 % ethanol, 1 × 45 min in a 1 % uranyl acetate solution in 70 % ethanol followed by 90 %, 100 % ethanol and propylene oxide for 2 × 10 min each. Incubate sequentially with 3:1, 1:1, 1:3 propylene oxide and Embed-812 mix, 30 min each and finally incubate overnight at room temperature in straight Embed-812. Sections including GPe and GPi were flat-embedded and baked in 60 °C oven for 72 h.

Following polymerization, the region of interest was checked employing low-magnification lens; using the point of a sharp scalpel the areas of interest (GPe and GPi) were cut out. The cut fragments were glued onto resin specimen blocks, previously polymerized, and stored at 4 °C. Using a Leica Ultracut R ultramicrotome thin sections of silver-gold color were collected on carbon-coated grids (150 mesh) and store until use.

Grids were examined using a digital Zeiss Libra 120 energy filter transmission microscope (EFTEM) operated at 80,000 kV.

### Statistical analyses

The intensity of CB_1_R and CB_2_R mRNA expression in CTB-labeled neurons was measured with bi-dimensional densitometry software available for the Zeiss 510 Meta CLSM. Briefly, a flat projection of each confocal stack obtained with the ×40 oil-immersion lens was generated for each channel showing CTB, CB_1_R or CB_2_R mRNA labeling. The number of pixels within a given region of interest (ROI) were counted at the single-cell level and normalized against the background staining. For each animal, the densitometry analysis was performed on approximately 80 CTB-labeled neurons. The only neurons considered as appropriate ROIs for densitometric analysis were those in which the nucleus was clearly visible. The means and standard deviations were then calculated and compared for each variable (CB_1_R mRNA and CB_2_R mRNA). The values across the two monkeys in each group were homogeneous, therefore considered as ‘statistically equivalent’ and thus the values were analyzed together. We assessed the statistical significance of the differences between the experimental groups using ANOVA tests followed by post hoc tests for multiple comparisons. All *p* values reported here are two-tailed and statistical significance was defined a priori at *p* = 0.05. Data analyses were performed using SPSS 15.0 (SPSS, Inc.).

## Results

### Expression of CB_1_R and CB_2_R mRNA transcripts in the GPi nucleus

**Fig. 1:**
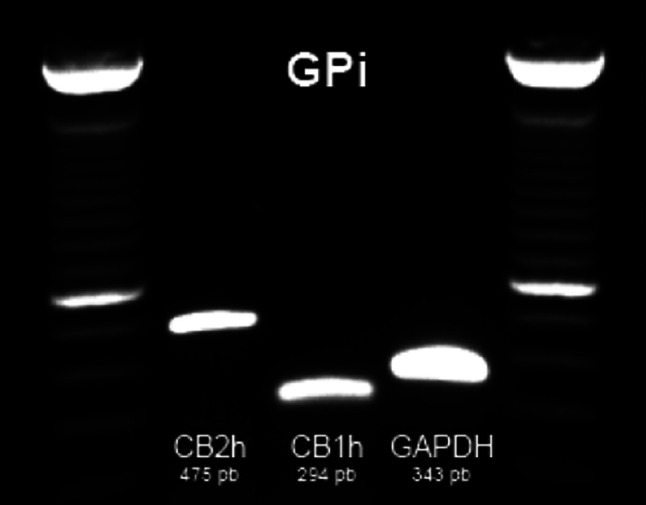
Detection by PCR amplification of CB_1_R and CB_2_R mRNA transcripts in the GPi. GAPDH mRNA was used as a positive control

**Fig. 2:**
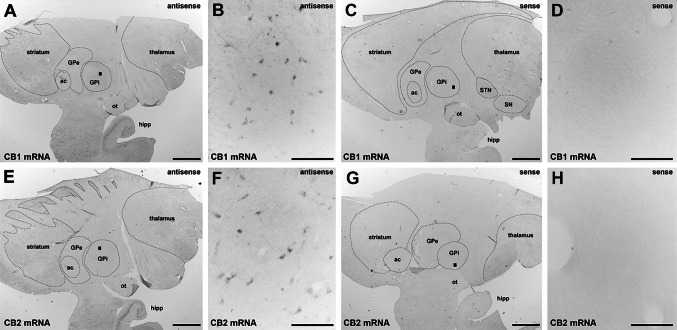
Detection of CB_1_R and CB_2_R mRNA using in situ hybridization. Using colorimetric in situ hybridization in a naïve primate, CB_1_R and CB_2_R mRNA (*panels* **a**, **b** and **e**, **f**, respectively) were detected in the GPi nucleus. The sense probes did not provide specific labeling of CB_1_R or CB_2_R mRNA (*panels* **c**, **d** and **g**, **h**, respectively). Even at low magnification, a lack of stain when using sense probes for CB_1_R and CB_2_R mRNA (*panels* **c** and **g**) was observed in the hippocampal formation, which was stained specifically when using antisense probes for both transcripts (**a** and **e**). *Scale bar* is 3,000 μm for *panels* **a**, **c**, **e** and **g** and 150 μm for insets **b**, **d**, **f** and **h**. *ac* anterior commissure, *GPe* external division of the globus pallidus, GPi internal division of the globus pallidus, *hipp* hippocampal formation, *ot* optic tract, *SN* substantia nigra, *STN* subthalamic nucleus

The presence of CB_1_R and CB_2_R mRNA transcripts in GPi was confirmed by PCR (Fig. 1) in samples from two naïve animals. Sense and antisense riboprobes for CB_1_R and CB_2_R mRNAs were generated and tested to confirm the presence of specific hybridization signals. Appropriate levels of gene expression were detected using antisense riboprobes, whereas hybridization with the riboprobe in the sense direction resulted in a complete lack of stain (Fig. 2).

### Co-expression of CB_1_R and CB_2_R mRNA in pallidothalamic projection neurons

**Fig. 3:**
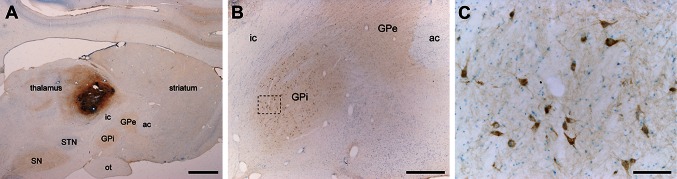
Retrograde CTB labeling of pallidothalamic-projecting neurons. CTB deposits were placed at the level of VA/VL thalamic nuclei in control, parkinsonian and dyskinetic monkeys. Following CTB injection in VA/VL nuclei (**a**), a large number of retrogradely labeled neurons were found throughout all territories of the GPi nucleus (**b**). *Panel* **c** shows an inset taken from *panel* B at a higher magnification. *Scale bar* is 3,000 μm in *panel* **a**, 1,000 μm in *panel* **b**, and 100 μm in *panel* **c**. *ac* anterior commissure, *GPe* external division of the globus pallidus, *GPi* internal division of the globus pallidus, *ic* internal capsule, *SN* substantia nigra, *STN* subthalamic nucleus, *ot* optic tract

Pallidothalamic-projecting neurons of naïve animals were unequivocally identified following the delivery of large deposits of CTB in the VA/VL thalamic nuclei in six primates (2 control, 2 parkinsonian and 2 dyskinetic). Tracer leakage through the needle tract was not observed in any of the CTB-injected monkeys (an example of an injection site is illustrated in Fig. 3). In all cases, following the delivery of CTB in the VA/VL, a large number of retrogradely labeled neurons was found in the ipsilateral GPi nucleus (Fig. 3) and substantia nigra *pars reticulata*, as well as in the pedunculopontine nucleus, bilaterally. A more moderate number of CTB-labeled neurons was observed in the ipsilateral subthalamic nucleus (Rico et al. 2010) and in the contralateral deep cerebellar nuclei.

**Fig. 4:**
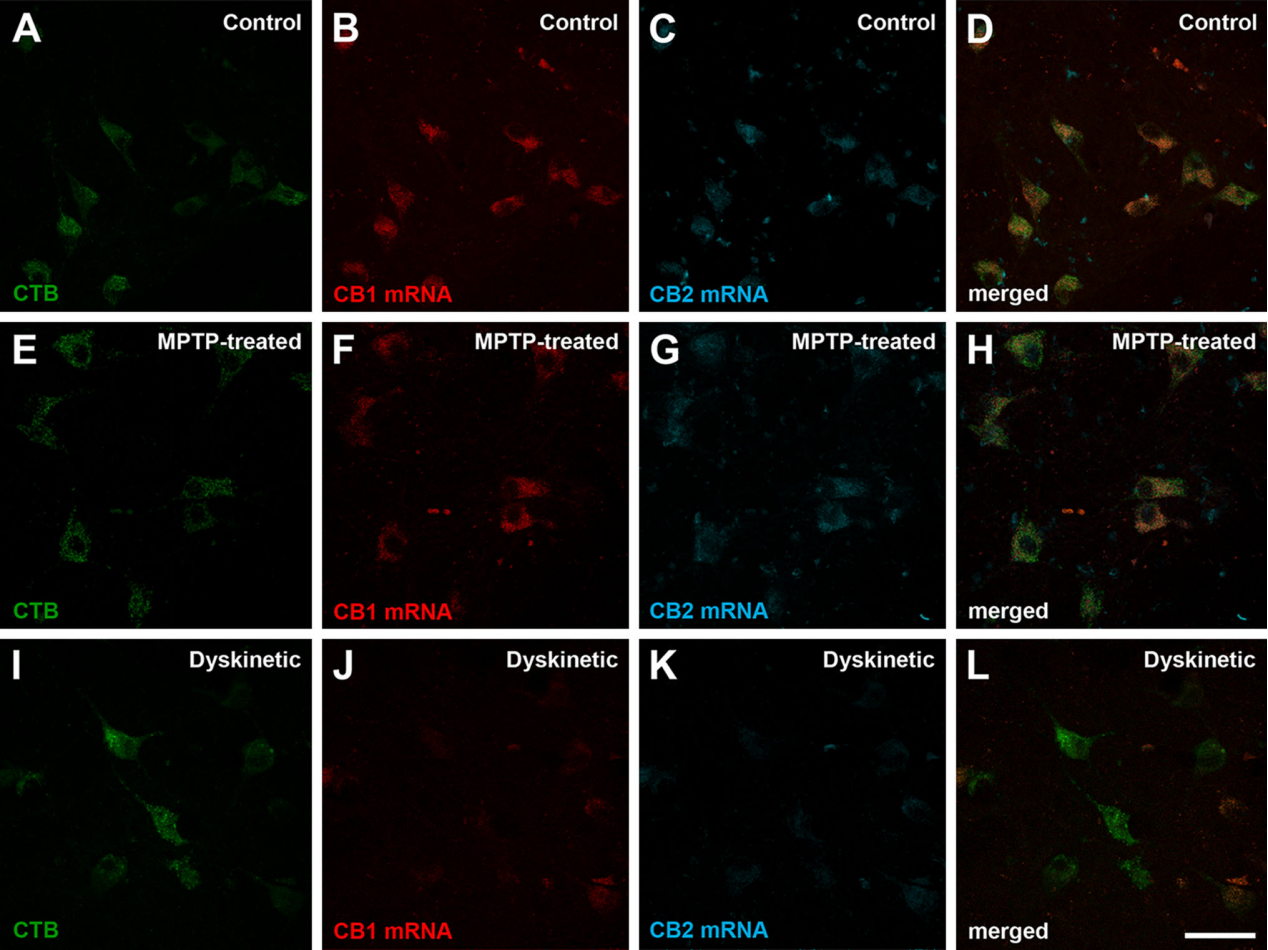
Co-expression of CB_1_R and CB_2_R mRNA in pallidothalamic neurons in control, parkinsonian and dyskinetic monkeys. Immunofluorescent detection of transported CTB combined with dual fluorescent in situ hybridization for the detection of CB_1_R and CB_2_R mRNA. All pallidothalamic projecting neurons (*green channel*) co-expressed CB_1_R (*red channel*) and CB_2_R (*blue channel*) mRNA. CB_2_R mRNA was expressed at lower levels than CB_1_R mRNA across all experimental conditions. Most importantly, there was a marked reduction in expression levels for both CB_1_R and CB_2_R mRNA transcripts in the dyskinetic state. *Scale bar* is 50 μm for all *panels*

The combination of dual fluorescent in situ hybridization together with the immunofluorescent detection of CTB enabled the unequivocal demonstration that all pallidothalamic-projecting neurons co-expressed both CB_1_R and CB_2_R mRNA, as seen in control, parkinsonian and dyskinetic monkeys (Fig. 4). Only a minimal fraction of CTB-labeled neurons (less than 1 %) did not show CB_1_R and CB_2_R mRNA transcripts, whereas a few CTB-unlabeled neurons displayed CB_1_R and CB_2_R mRNA co-expression.

### Quantification of CB_1_R and CB_2_R mRNA expression levels

**Fig. 5:**
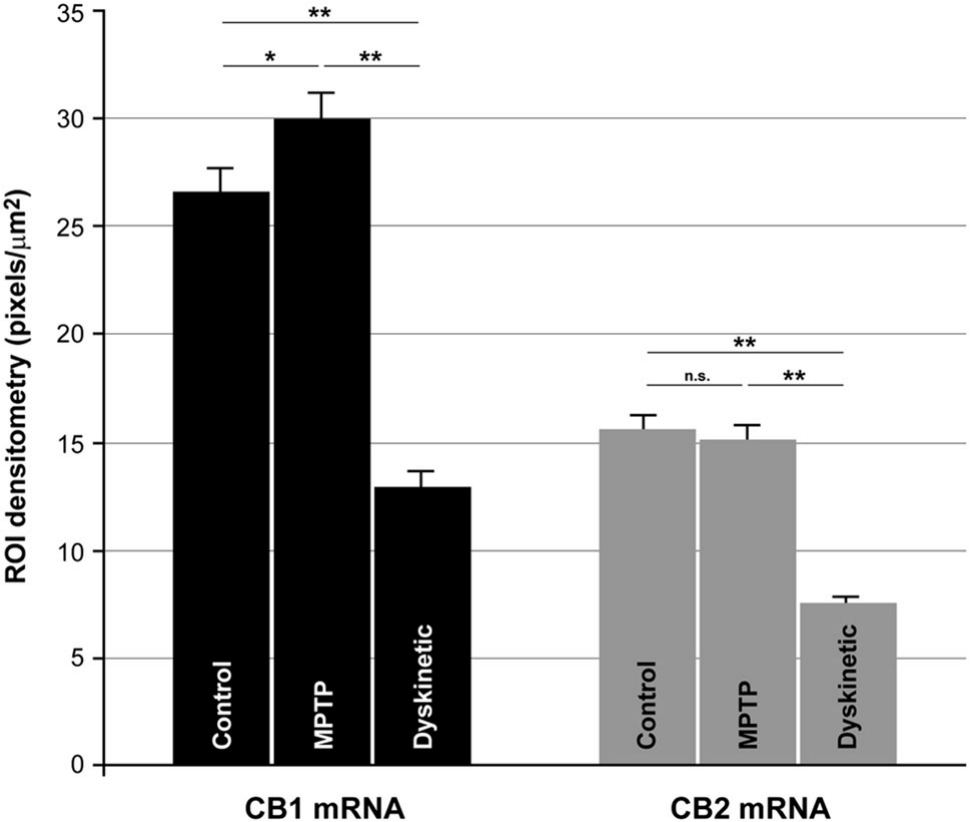
Quantification of CB_1_R and CB_2_R mRNA expression levels in control, parkinsonian and dyskinetic monkeys. Histograms show the mean values of the expression levels for each transcript of interest across the experimental groups analyzed. Densitometries were carried out at the single-cell level by counting the number of pixels per μm^2^ within a given region of interest (ROI). Measurements were taken from a minimum of 80 neurons per monkey. Differences in CB_1_R mRNA expression levels were statistically significant between control and MPTP-treated animals (*p* = 0.021), control and dyskinetic monkeys (*p* < 0.001) and MPTP-treated and dyskinetic (*p* < 0.001). The mean difference values for CB_2_R mRNA were statistically significant between control and dyskinetic animals (*p* < 0.001) and between MPTP and dyskinetic groups (*p* < 0.001). Differences in CB_2_R mRNA expression levels between control and MPTP-treated monkeys were not significant (n.s., *p* = 0.946)

CB_1_R mRNA levels were consistently higher than CB_2_R mRNA levels in all three experimental groups (Fig. 5). Variance analysis showed intra-group homogeneity. Given that the variance value for either CB_1_R or CB_2_R was not homogeneous across the three experimental groups, the Tamhane’s post hoc test was used for intergroup comparisons. Using this test to compare CB_1_R mRNA values, the mean differences between control and MPTP (*p* = 0.021), control and dyskinetic (*p* < 0.001) and MPTP and dyskinetic groups (*p* < 0.001) were statistically significant. The mean differences of CB_2_R values between groups were statistically significant between control and dyskinetic (*p* < 0.001) and between MPTP and dyskinetic groups (*p* < 0.001); however no difference that was statistically significant was found between control and MPTP monkeys (*p* = 0.946). Dyskinetic monkeys displayed a marked downregulation of both CB_1_R and CB_2_R mRNA expression with respect to the levels found both in control and MPTP-treated monkeys (Fig. 5).

### Presence of CB_1_R–CB_2_R heteromers in transfected cells: BRET analysis

**Fig. 6:**
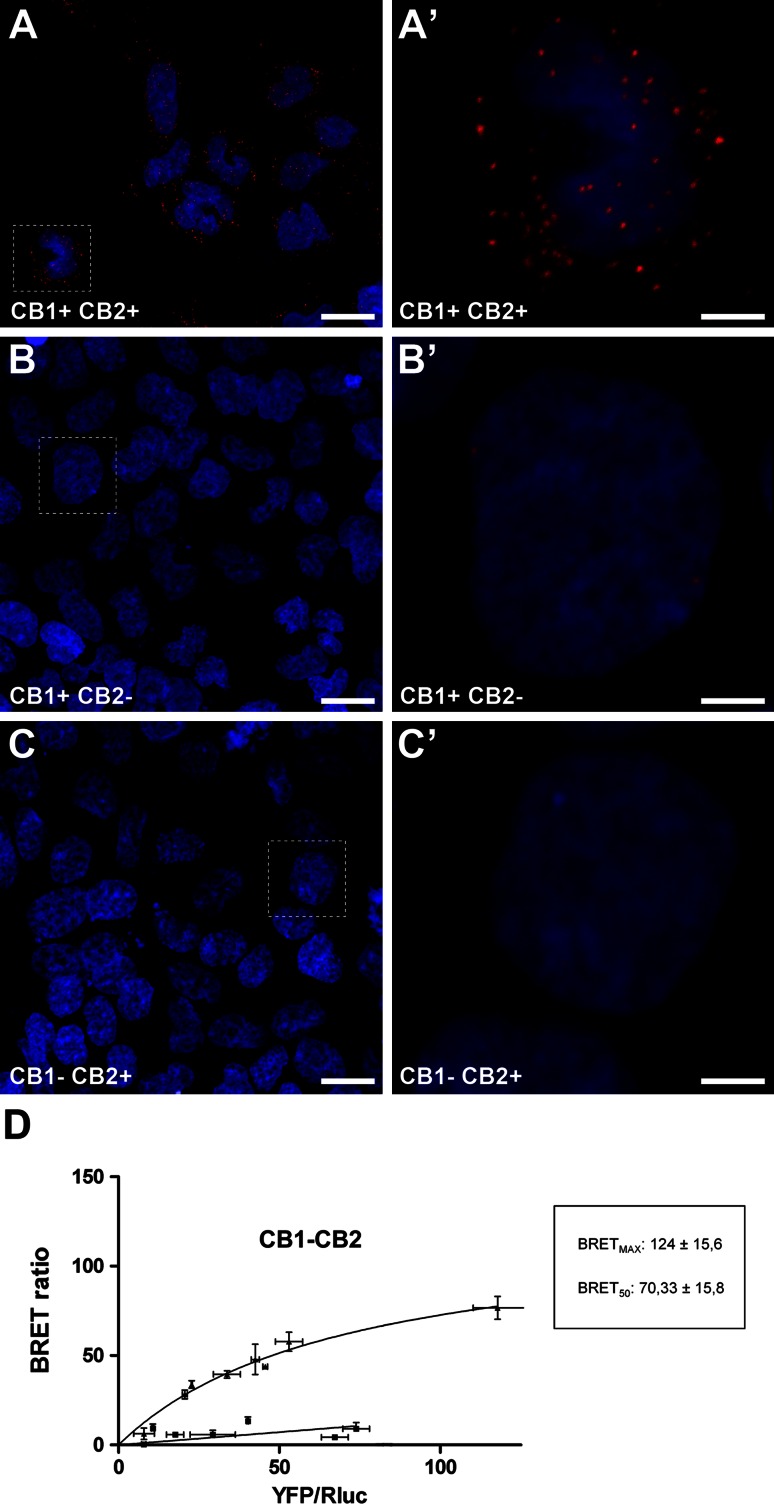
Detection of CB_1_–CB_2_ receptor heteromers in HEK-transfected cells. The specificity of the in situ proximity ligation assay (PLA) was tested using HEK-293T cell lines transiently transfected with the cDNAs of CB_1_R and CB_2_R (**a**, **a**’), with CB_1_R only (**b**, **b**’) or with CB_2_R only (**c**, **c**’). Cells were processed for PLA stain according to the guidelines issued by the manufacturer. Only when the two receptors were present and in close proximity were CB_1_R–CB_2_R heteromers detected as a punctuate fluorescent signal by confocal microscopy. Since receptors are recognized by primary antibodies linked to different DNA chains (a plus and a minus), CB_1_R–CB_2_R receptor heteromers were only detected in HEK cells transfected with both cDNAs but not in cells transfected only with either CB_1_ or CB_2_ cDNAs. *Scale bar* is 20 μm for *panels* **a**, **b** and **c**, and 5 μm for insets. **d** BRET saturation experiments showing CB_1_R–CB_2_R heteromerization were performed using cells transfected with 1 µg of cDNA corresponding to CB_1_R–Rlu*c* and increasing amounts of cDNA (0–3 µg cDNA) corresponding to CB_2_R-YFP (*triangles*). As a negative control, cells were also transfected with cDNA corresponding to CB_1_R–Rlu*c* (1 µg) and to D_4,2_R–YFP (0–4 µg cDNA) (*squares*). Both fluorescence and luminescence for each sample was measured before every experiment to confirm similar donor expressions (approximately 100,000 bioluminescence units) while monitoring the increase in acceptor expression (100–70,000 net fluorescence units). The relative amount of BRET is given as the ratio between the net fluorescence of the acceptor (YFP) and the luciferase activity of the donor (*Rluc*). BRET data are expressed as mean ± s.e.m. of 4–8 different experiments grouped as a function of the amount of BRET acceptor

Evidence showing that CB_1_Rs and CB_2_Rs form heteromers in HEK-293T transfected cells was reported recently (Callen et al. 2012). Here we took advantage of this finding to perform control experiments showing the specificity of the PLA technique. HEK-293T cells transiently expressing CB_1_R, CB_2_R or both receptors were processed using the PLA technique. As expected, CB_1_R–CB_2_R heteromers were only identified in HEK-293T cells co-transfected with both receptors, whereas cells containing only CB_1_Rs or CB_2_Rs always lacked a positive PLA product (Fig. 6a, b, c). Furthermore, the presence of molecular interactions within CB_1_R–CB_2_R heteromers was confirmed by BRET measurements taken from co-transfected HEK-293T cells. As shown in Fig. 6d, the BRET signal increased as a hyperbolic function of the amount of CB_1_R–YFP expressed, whereas the negative control made of CB_1_R–Rluc and D_4,2_R-YFP resulted in a low and linear BRET saturation curve.

### Presence of CB_1_Rs, CB_2_Rs and CB_1_R–CB_2_R heteromers in pallidothalamic neurons

**Fig. 7:**
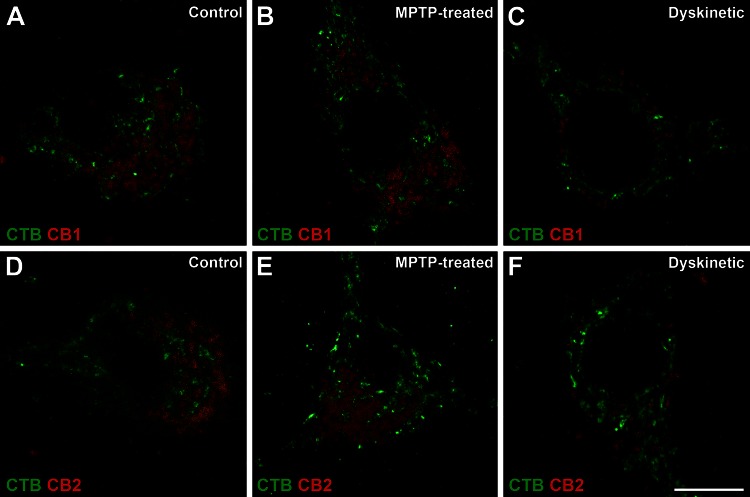
Presence of CB_1_Rs and CB_2_Rs in pallidothalamic-projecting neurons of control, parkinsonian and dyskinetic monkeys. A modified version of the PLA technique enabled the visualization of single cannabinoid receptors (*red dots*) within CTB-labeled pallidothalamic projection neurons (*green marker*). *Panels* **a**–**c** show CB_1_Rs in pallidal efferent neurons of control (**a**), MPTP-treated (**b**) and dyskinetic (**c**) monkeys. *Panels* **d**, **e** illustrate CB_2_Rs in pallidal efferent neurons of control (**d**), MPTP treated (**e**) and dyskinetic (**f**) monkeys. The number of both types of receptors was clearly reduced in the dyskinetic state. *Scale bar* is 10 μm in all *panels*

Using a modified version of the PLA protocol the presence of CB_1_Rs or CB_2_Rs was assessed within CTB-labeled pallidothalamic projection neurons (Fig. 7). Obtained results showed that both cannabinoid receptors are expressed in pallidothalamic neurons and a qualitative analysis of the expression levels for either CB_1_Rs or CB_2_Rs showed a marked decline in both receptors in dyskinetic animals, in keeping with what was observed at the mRNA level. These results demonstrate that CB_1_R and CB_2_R mRNA transcripts observed with in situ hybridization are ultimately translated into related proteins and similar qualitative changes in mRNA and protein levels were observed in dyskinetic animals. The results also showed that the receptors were located in the cellular somata of projection neurons. Upon demonstrating that the two receptors were indeed synthesized, determining the presence of heteromer expression was the next logical step.

**Fig. 8:**
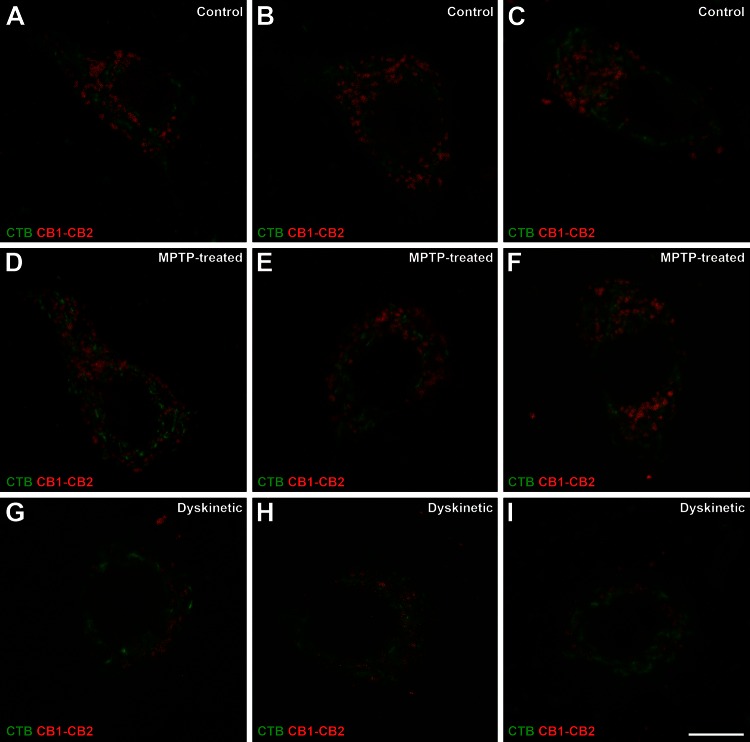
CB_1_R–CB_2_R heteromers in pallidothalamic-projecting neurons of control, parkinsonian and dyskinetic monkeys. Illustrative examples of identified projection neurons (CTB-labeled; *green*) taken from control (**a**–**c**), MPTP-treated (**d**–**f**) and dyskinetic (**g**–**i**) monkeys. Each red dot represents one CB_1_R–CB_2_R receptor heteromer. *Scale bar* is 10 μm in all *panels*

**Fig. 9:**
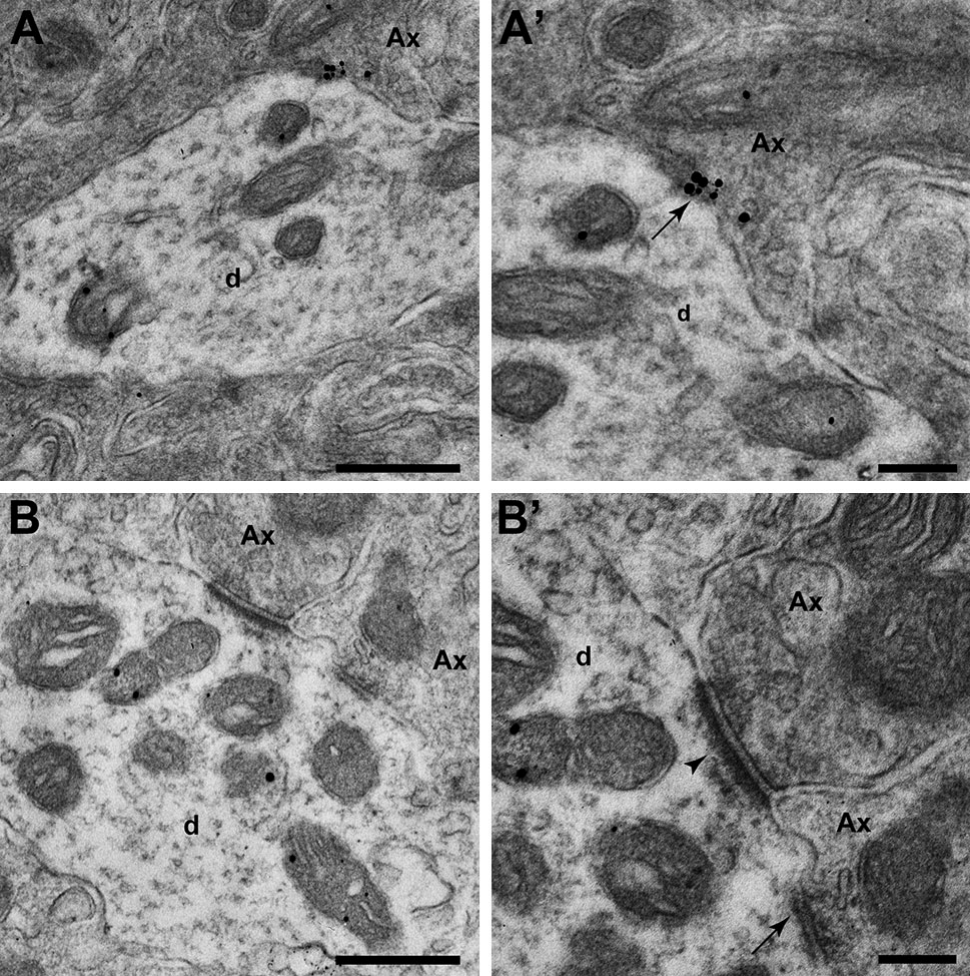
Ultrastructural localization of CB_1_R–CB_2_R heteromers in the GPe (**a**, **a**’) and GPi (**b**, **b**’) nuclei using the PLA technique. **a**, **a**’ Electron micrograph showing CB_1_R–CB_2_R heteromers located in the pre- (Ax) and postsynaptic elements (d) in GPe. (**a**’) Inset taken from (**a**) at higher magnification showing that cannabinoid receptor heteromers are found in both the pre- and postsynaptic membranes in a symmetric synapse (*arrow*). **b**, **b**’ At the level of the GPi nucleus, cannabinoid receptor heteromers are confined to postsynaptic locations (d). (**b**’) Inset taken from (**b**) at higher magnification showing a GPi dendrite (d) simultaneously receiving one symmetrical (*arrow*) and one asymmetrical synapses (*arrowhead*). *Scale bar* is 500 nm for panels **a** and **b**, and 200 nm for insets **a**' and **b**'

We showed that CB_1_R–CB_2_R heteromers are present in pallidothalamic projection neurons (Fig. 8). Qualitative analysis of the relative levels of CB_1_R–CB_2_R heteromers in these neurons revealed that, while there were no discernable differences between control and parkinsonian monkeys, there was a marked reduction in dyskinetic monkeys (Fig. 8). It is worth noting that this reduction in CB_1_R–CB_2_R heteromers observed in dyskinetic monkeys with the PLA technique mimics the marked decrease in CB_1_R and CB_2_R mRNA expression levels observed using dual fluorescent in situ hybridization assays. Moreover, most of the CB_1_Rs, CB_2_Rs and CB_1_R–CB_2_R heteromers were observed in subcellular locations instead of in the plasma membrane (Figs. 7, 8). This is in keeping with the earlier reports providing ultrastructural evidence on the presence of CB_1_Rs in somatodendritic compartments of rat striatal neurons (Rodríguez et al. 2001) as well as in putative GABAergic interneurons of the monkey prefrontal cortex (Eggan and Lewis 2007). Indeed, Leterrier et al. (2006) reported that approximately 30 % of CB_1_Rs were located in endosomes, 50 % in intracellular, nonendosomal locations and only between 10 and 20 % of receptors were observed in the plasma membrane. These data were corroborated here following the ultrastructural detection of PLA-stained material for CB_1_R–CB_2_R heteromers. The study of GPe sections showed the presence of CB_1_R–CB_2_R heteromers in both pre- and postsynaptic membranes of symmetric synapses (Fig. 9a, a’). Meanwhile, the study of GPi sections showed the presence of CB_1_R–CB_2_R heteromers mainly in postsynaptic locations and the lack of inmunoreactivity for CB_1_R–CB_2_R heteromers in axon terminals, those comprising both symmetric and asymmetric synapses (Fig. 9b, b’).

## Discussion

### Technical considerations

Here we demonstrate the co-expression of CB_1_R and CB_2_R mRNA transcripts within pallidothalamic-projecting neurons in the monkey, and provide quantitative measurements of the changes in mRNA expression levels across different clinical conditions. The main caveat of measuring levels of mRNA transcripts is that they may not always correlate with receptor protein levels (Sossin and DesGroseillers 2006). Previous studies have shown, however, that differences in CB_1_R mRNA expression levels are matched by differences in CB_1_R immunoreactivity and CB_1_R ligand binding (Herkenham et al. 1991a, b; Mailleux and Vanderhaeghen 1992; Egertová and Elphick 2000; Julian et al. 2003). Functionally, it has been demonstrated that developmental changes in postsynaptic suppression of excitation in pyramidal neurons of the prefrontal cortex are CB_1_R-mediated and these variations are paralleled by changes in CB_1_R mRNA levels (Heng et al. 2011).

Another matter of concern is represented by the fact that commercially available cannabinoid receptor antibodies are not all created equal (Grimsey et al. 2008). In brief, antibodies for CB_1_R are made against either the N-terminal or the C-terminal and it is clear that a single antibody is unlikely to detect all receptor species. Indeed, antibodies directed towards different regions of CB_1_R may be expected to yield different staining patterns. Here we used a CB_1_R antibody directed against the N-terminal (from Thermo Scientific), according with earlier descriptions dealing with CB_1_R distribution in the primate brain (Ong and Mackie 1999; Eggan and Lewis 2007). At the level of the GPi nucleus, Ong and Mackie (1999) reported the presence of scattered large diameter neurons, together with very few immunoreactive fibers in the neutrophil, results that are very much in keeping with those reported here. Furthermore, it is also worth noting that extensive CB_1_R immunoreactivity was described in GABAergic striatopallidal boutons in rodents (Mátyás et al. 2006). In this regard, comparing the globus pallidus in rodent and primates is often a source of misinterpretations, since the rodent globus pallidus makes reference to the lateral globus pallidus (LGP; external pallidus in monkeys, GPe), whereas the rodent equivalent to the internal division of the globus pallidus (GPi) in monkeys is represented both by the entopeduncular nucleus (ENT) and the substantia nigra pars reticulata (SNr). Because the ENT nucleus is made up of a very few number of neurons intermingled within the fiber bundles of the internal capsule, the SNr is considered as the main basal ganglia output nucleus in rodents. In other words and for comparison purposes, the globus pallidus in rodents is the equivalent structure to the primate GPe, and the same applies to the rodent SNr and the primate GPi. In this regard, the presence of CB_1_R immunostaining was mainly found in striatopallidal terminals reaching the globus pallidus in rodents, whereas in the SNr the majority of axon terminals forming symmetrical synapses were CB_1_R negative (Mátyás et al. 2006). In an attempt to clarify this issue, we have tested a different anti-CB_1_R antibody (directed towards the C terminus of the receptor, known as the “Watanabe’s antibody” and purchased from Frontiers Science, Japan). Using this antibody in the monkey brain resulted in intense GPi neuropil labeling. When compared with the nicely stained fibers and terminals observed in the cerebral cortex and hippocampal formation, the GPi stain is diffuse and therefore not enough accurate to properly disclose CB_1_R-labeled fibers and terminals (data not shown).

PLA results constitute the first description of CB_1_R–CB_2_R heteromers in pallidothalamic projection neurons in the monkey. The correlation between mRNA levels and the amount of CB_1_R–CB_2_R heteromers detected by PLA suggest that mRNAs for cannabinoid receptors in GPi neurons are readily translated into protein. Although data gathered from the PLA assays were merely qualitative, observed changes fit with the data obtained from in situ hybridization experiments. It is worth noting that it is not technically possible to provide data on total CB_1_R or CB_2_R levels in natural tissues and therefore we could not address whether receptor heteromers were the only cannabinoid signaling unit. Experiments carried out in rodents have shown that CB_2_Rs may downregulate CB_1_R-mediated signaling when forming heteromers with CB_1_Rs (Callen et al. 2012). Indeed, a shift from CB_1_R to CB_1_R–CB_2_R signaling could have significant functional implications. In other words, the transcribed mRNA gives rise to monomers/homomers and to heteromers. Although the total number of CB_1_Rs and CB_2_Rs could be estimated using total membrane preparations of GPi homogenates, it should be noted that data gathered from this technique do not necessarily reflect the level of receptors on the cell surface, which is the place where endocannabinoids mainly interact with their related receptors. Moreover, receptor quantification by means of radioligand binding competition assays may not result in fully reliable data as the affinity constants vary when a given receptor is forming different heteromers when comparing control versus “diseased” animal models. The proportion of receptors forming or not heteromers at any given time is dynamic and may change in response to activity or pathological states (Gonzalez et al. 2012). The combination of mRNA and heteromer detection across all experimental groups supports this idea and, interestingly, whereas no noticeable difference in the number of CB_1_Rs, CB_2_Rs and CB_1_R–CB_2_R heteromers was found between control and parkinsonian monkeys, a marked decreased was noticed in dyskinetic monkeys.

### Presence of cannabinoid receptors and receptor heteromers in pallidothalamic neurons

Data gathered using in situ hybridization cannot provide information about pre- or postsynaptic distribution of CB_1_Rs and CB_2_Rs. These receptors may be transported anterogradely to distal axon terminals (presynaptic distribution), remain in close vicinity to the place of synthesis to be incorporated in the plasma membrane in cell somata and dendrites (postsynaptic distribution), or a combination of the two. Results obtained with the PLA technique do provide, however, information concerning the distribution of cannabinoid receptors in the GPi. Here, CB_1_Rs, CB_2_Rs and CB_1_R–CB_2_R heteromer complexes were identified in the cell bodies of pallidothalamic projection neurons (CTB-labeled).

Obtained data were also confirmed using electron microscopy, describing for the first time CB_1_R–CB_2_R heteromers in primate GPi. Our results showed the presence of heteromers in postsynaptic elements in the GPi. For comparison purposes, the GPe was also studied, where we have found a different inmunoreactive pattern: unlike in the GPi, at the GPe level CB_1_R–CB_2_R heteromers are mainly found in both pre- and postsynaptic membranes. Previous studies in other cerebral areas in mouse, rat, monkeys or humans have shown that CB_1_Rs are mainly present in the presynaptic elements (Katona et al. 1999, 2000; Eggan and Lewis 2007; Mátyás et al. 2006; Lafourcade et al. 2007; Chiu et al. 2010; Puente et al. 2010; Reguero et al. 2011) while other studies had describe the presence of CB_1_R in pre- and postsynaptic elements, even located in neuronal somas (Ong and Mackie 1999; Rodríguez et al. 2001; Pickel et al. 2004; Wilson-Poe et al. 2012). Since the presence of CB_1_R–CB_2_R heteromers in GPi in primates was not previously described, we consider that the absence of those heteromers in the presynaptic element is plausible. Regarding methodological considerations at the ultrastructural level, we have considered inmunoreactive CB_1_R–CB_2_R profiles when even only one gold particle was present, since the background labeling was minimal and in keeping with earlier studies showing that even one gold particle in small profiles can represent an important density of labeling (Wang and Pickel 2002; Pickel et al. 2004). Gold silver labeling method shows a lower relatively sensitivity than the peroxidase method, however gold-silver labeling does permit a more precise subcellular localization of inmunoreactivity.

Endocannabinoids activate CB_1_Rs via a retrograde signaling process in which the compounds are released from postsynaptic neuronal elements, travelling back to the presynaptic terminal to act on pre- and perisynaptic receptors. This mechanism has been implicated in short-term synaptic depression, including suppression of excitatory or inhibitory transmission (see Lovinger 2008, for review). A relevant question is why the two cannabinoid receptors subtypes are co-expressed in the same projection neurons. Both receptors are G-protein-coupled receptors (GPCRs) linked to G_i/o_ proteins (Bayewitch et al. 1995; Gonsiorek et al. 2000; Shoemaker et al. 2005), i.e. negatively coupled to adenylyl cyclase (Demuth and Molleman 2006). Further, the endocannabinoid 2-arachidonoylglycerol is a full endogenous agonist of both CB_1_Rs and CB_2_Rs, although CB_2_Rs have a higher sensitivity to this molecule (Atwood et al. 2012). While the two receptors behave similarly from a pharmacological point of view, receptor heteromerization suggest a postsynaptic signaling unit constituted by the heteromer that likely conveys a specific signal in pallidothalamic neurons. It is already accepted that GPCR heteromers are functionally distinct units and not a mere assembly of two receptors with independent functions (Ferré et al. 2009). Similar examples of heteromers for the same subfamily include opioid (Constantino et al. 2012), dopamine (Hasbi et al. 2009; Perreault et al. 2012) and adenosine (Ciruela et al. 2006) receptors, among others (reviewed in Hiller et al. 2013). The role of CB_1_R–CB_2_R heteromers in basal ganglia output signal modulation is a matter of speculation but the prediction would be that some of the conflicting data on comparing in vitro cell pharmacology with behavioral responses to endocannabinoids or to synthetic ligands could be attributed to the occurrence of CB_1_R–CB_2_R heteromers in pallidothalamic neurons.

### Cannabinoid receptors in the parkinsonian state

Increases in CB_1_R mRNA expression levels (Mailleux and Vanderhaeghen 1993; Romero et al. 2000), and in CB_1_R ligand binding (Lastres-Becker et al. 2001) have been reported in parkinsonian animals. An increase in presynaptic CB_1_R levels in corticostriatal neurons would lead to a reduction in glutamate release (Gerdeman and Lovinger 2001; Brown et al. 2003), possibly representing a compensatory mechanism. The present study shows a slight increase in CB_1_R mRNA transcripts within GPi neurons of MPTP-treated monkeys. CB_1_Rs may be transported through GABAergic terminals to the thalamus to reduce GABA release in an attempt to decrease over-inhibition of the thalamus. Alternatively, this modest increase could simply be a secondary effect to the increase in subthalamic glutamatergic afferents to GPi, whereby an increase in glutamatergic receptor activation can stimulate an increase in CB_1_R mRNA synthesis (Mailleux and Vanderhaeghen 1994). However this possibility would not fit with the subcellular localization of the receptors in pallidothalamic neurons. Moreover, ultrastructural evidence of CB_1_Rs located in the somatodendritic compartment of striatal neurons in rats has been reported elsewhere (Rodríguez et al. 2001). Indeed, there are also available evidences showing that CB_1_Rs are associated to the Golgi apparatus and rough endoplasmic reticulum within GABAergic interneurons of the macaque prefrontal cortex (Eggan and Lewis 2007). In this regard, Leterrier et al. (2006) reported that approximately 30 % of CB_1_Rs are located in subcellular endosomal compartment and only between 10 and 20 % on the plasma membrane, the rest being intracellular, nonendosomal receptors. Concerning CB_2_Rs, the results reported here cannot be compared with other in the literature due to the lack of data describing expression levels in parkinsonian states. Further experiments will be required to properly assess the subcellular localization of CB_1_Rs, CB_2_Rs and CB_1_R–CB_2_R heteromers, as well as the potential changes in receptor distribution following different experimental conditions. Upregulation of CB_2_Rs in conditions of striatal degeneration in glial cells has been described in Huntington’s disease (Sagredo et al. 2009), however it is not yet clear whether CB_2_Rs are upregulated in glial cells in response to neuronal damage in PD (reviewed in Fernandez-Ruiz et al. 2007, 2008). The small increase in CB_1_R mRNA in parkinsonian macaques did not translate into a higher quantity of CB_1_R–CB_2_R heteromers, suggesting that either CB_2_Rs are a limiting factor in the formation of these heteromers or that the increase in CB_1_Rs was not sufficient to significantly affect the number of CB_1_R–CB_2_R heteromers formed.

### Cannabinoid receptors in the dyskinetic state

With regard to data from dyskinetic animals, both CB_1_R and CB_2_R mRNAs synthesized in the GPi decreased with respect to the levels found in control and parkinsonian monkeys. A marked decrease in CB_1_Rs, CB_2_Rs and CB_1_R–CB_2_R heteromers was also found. These changes in expression levels and heteromer formation may be compensatory in an attempt to reverse increased neuronal activity to a state of normality. A decrease in CB_1_Rs and/or CB_2_Rs located on pallidothalamic neurons may lower the threshold for firing underactive GPi neurons by glutamate released from the subthalamic nucleus.

There is evidence for a downregulation of CB_1_Rs in the early stages or presymptomatic states of PD (Garcia-Arencibia et al. 2009) and the dyskinetic state may emulate this phenomenon. Although one study did find an increase in CB_1_R mRNA levels in the striatum in 6-OHDA lesioned rats chronically treated with levodopa (Zeng et al. 1999), levodopa has since then been found to consistently reverse both the elevation of endocannabinoid levels (Maccarrone et al. 2003; van der Stelt et al. 2005) and the PD-related increase in CB_1_R density and binding (Lastres-Becker et al. 2001). Both CB_1_R agonists (Ferrer et al. 2003; Gilgun-Sherki et al. 2003; Segovia et al. 2003; Fernandez-Ruiz et al. 2007; Morgese et al. 2007, 2009) and antagonists (Segovia et al. 2003; van der Stelt et al. 2005) show anti-dyskinetic activity in MPTP-treated primates and 6-OHDA lesioned rats. This apparent paradox may result from the presence of CB_1_Rs in both excitatory and inhibitory synapses within basal ganglia circuits and/or from the pre- and postsynaptic expression of CB_1_R-containing heteromers. Finally, it is worth noting that a recent study on GPCR heteromers made of adenosine 2A, CB_1_ and dopamine D_2_ receptors in macaques also showed that the chronic treatment with levodopa disrupts all these types of heteromers at the level of the caudate nucleus (Bonaventura et al. 2014).

### Concluding remarks

The presence of CB_1_R–CB_2_R heteromers in pallidothalamic neurons adds a new dimension to their role in basal ganglia function. Determining the precise function of CB_1_R–CB_2_R heteromers and elucidating the way in which these receptors modify neuronal signaling in the GPi will pave the way for the discovery of specific drugs that may either reduce GPi overactivity in the parkinsonian state or provide more effective management of dyskinesia.
